# Dynamic Characteristics of Micro-Beams Considering the Effect of Flexible Supports

**DOI:** 10.3390/s131215880

**Published:** 2013-11-25

**Authors:** Zuo-Yang Zhong, Wen-Ming Zhang, Guang Meng

**Affiliations:** State Key Laboratory of Mechanical System and Vibration, School of Mechanical Engineering, Shanghai Jiao Tong University, Shanghai 200240, China; E-Mails: zhongzuoyang123@163.com (Z.-Y.Z.); gmeng@sjtu.edu.cn (G.M.)

**Keywords:** equivalent stiffness, flexible supports, micro-beams, resonance frequency shift, dynamics

## Abstract

Normally, the boundaries are assumed to allow small deflections and moments for MEMS beams with flexible supports. The non-ideal boundary conditions have a significant effect on the qualitative dynamical behavior. In this paper, by employing the principle of energy equivalence, rigorous theoretical solutions of the tangential and rotational equivalent stiffness are derived based on the Boussinesq's and Cerruti's displacement equations. The non-dimensional differential partial equation of the motion, as well as coupled boundary conditions, are solved analytically using the method of multiple time scales. The closed-form solution provides a direct insight into the relationship between the boundary conditions and vibration characteristics of the dynamic system, in which resonance frequencies increase with the nonlinear mechanical spring effect but decrease with the effect of flexible supports. The obtained results of frequencies and mode shapes are compared with the cases of ideal boundary conditions, and the differences between them are contrasted on frequency response curves. The influences of the support material property on the equivalent stiffness and resonance frequency shift are also discussed. It is demonstrated that the proposed model with the flexible supports boundary conditions has significant effect on the rigorous quantitative dynamical analysis of the MEMS beams. Moreover, the proposed analytical solutions are in good agreement with those obtained from finite element analyses.

## 1. Introduction

Micro-beams [1–3] are widely used as the key components of diverse sensing and actuation systems [4–6]. Their relatively simple geometries make them very advantageous [7–9], both from a design and microfabrication point of view. In the wide range of applications [10–13], ranging from the mean residual stress measurement, microscopy, mass flow sensors to bio-medical or DNA analysis, the sensing mechanism depends upon the sensitivity or response of the MEMS beam to some applied excitation. They accurately predict the dynamic features of the device such as its natural frequencies and forced-vibration response [14–16].

Microfabrication methods and limitations can lead to boundary support conditions for suspended MEMS beams that are not rigidly clamped [17,18]. Real system behavior may deviate from the idealized support conditions [19–23]. Under the ideal conditions, a beam connected at ends to rigid supports by pins is modeled assuming that the deflections and moments at the supports are zero. In reality, fabricating MEMS beams of ideal anchors with the precise intended design dimensions is hard to achieve practically due to fabrication imperfections, such as undercuts near anchors and initial deformation of MEMS beams due to residual stresses. Small deviations from the ideal conditions indeed occur at the ends [24,25]. The concept of non-ideal boundary conditions has been proposed to be applied to micro/nano-resonator systems [11,26]. Alkharabsheh and Younis [27] demonstrated that non-ideal boundary conditions can have significant effect on the qualitative static or dynamic behavior of MEMS beams, which includes lowering the natural frequencies from the expected range of operation and causing unpredictable dynamic pull-in. In this regard, support boundary characterization is important in the applications such as flexible optical waveguides [28] and AFM cantilever probes [29]. In the AFM tapping mode (dynamic mode), non-classical boundary supports have a big influence on the frequency response of the AFM probe. Furthermore, Boyaci *et al.* [21,30,31] reported that the non-ideality causes a shift in the frequency-response. By shifting the frequency-response curve, a system under resonance may be brought to a safer operating condition.

Hence, the boundary support conditions need to be theoretically quantified [32], and experimentally validated [33]. The numerical results of Mariani *et al.* [34] showed that the acceleration at sensor anchors couldn't be considered an objective indicator for drop severity. Instead, accurate analyses at a sensor level were necessary to illustrate how MEMS could fail because of drops. For flexible supports, several approaches have been followed to model the non-ideal boundaries conditions. Most researchers have attempted to solve the complete beam and supports structure using FEM [10,11,35–37]. Meanwhile, Mariani [38] proposed correction factors to be used in the analytical expressions (which neglect the compliance of the supports) that were obtained by comparing a linear model and FEM results. The information provided by the testing also provides feedback in understanding the effect of the correction factors.

In accordance, the boundaries are assumed to allow small deflections and moments [21,27,39]. Another approach that has been used by Boyaci *et al.* [21,30,31] and Ghayesh *et al.* [40] is to model the non-ideal boundary supports by introducing small deflection and moment as perturbation parameters in analytical models. The effect of the non-ideal boundary conditions is analyzed together with the non-linear effects. Spring elements have been added to the edges of the microstructures to model their compliant supports in the analytical modeling of Rinaldi *et al.* [41–45], which include normal, tangential and torsion springs. However, the values of spring constants [42] which are usually used to model the supports were obtained by comparing a linear elastic model or FEM results. In addition, due to the difficulties in directly measuring forces and other device parameters at the micro-scale, the spring constant of MEMS beams couldn't be extracted accurately from the dynamic response of the device. Esmailzadeh *et al.* [46] developed a feed-forward back-propagation artificial neural network which was used instead of the analytical solution. The performance of their neural network was evaluated for different values of parameters to save computation time.

In this paper, a rigorous theoretical solution is presented for the case of flexible supports of microbeams. Equivalent deformation in the normal and tangential direction at the boundary of the microbeam were formulated by Boussinesq's and Cerruti's displacement equations [47–49] due to a concentrated force acting on the surface of a semi-infinite elastic body. Then the tangential and rotational equivalent stiffness equations were separately derived by employing the principle of energy equivalence. The solutions described in this paper deal with the fundamental theoretical problem based on the classical elastic mechanics theory, which fills in the gap between the above three approaches for modeling the flexible supports of MEMS beams. The advantage of the proposed solution is that no approximated displacement and force fields are introduced during the derivation. The closed-form solution derived by the method of multiple timescales provides direct insight into the relationship between the boundary conditions and vibration characteristics of the system.

## 2. The Equivalent Stiffness

### 2.1. The Tangential Equivalent Stiffness

The displacement of any point (*x*, *y*) in the tangential direction formulated by Cerruti's displacement equations [47–49] due to a concentrated force *Q* acting on the surface of a semi-infinite elastic body is:

(1) $$\hat{u}(x,y)=\frac{1+v_{s}}{\pi E_{s}}\frac{Q}{\sqrt{x^{2}+y^{2}}}\left((1-v_{s})+v_{s}\frac{x^{2}}{x^{2}+y^{2}}\right)$$

where *E_s_*, *v_s_* are the Young's modulus and Poisson's ratio of the supports material. Utilizing the superposition principle and the energy equivalence principle, the tangential equivalent stiffness of a rectangle area (*w* × *b*) with the uniform vertical load *q* acted on had been derived as:

(2) $$K_{T}=\frac{\text{qwb}}{\bar{u}}=\frac{q^{2}w^{2}b^{2}}{2U_{t}}=\frac{\pi E_{s}w^{2}b^{2}}{(1-v_{s}^{2})H_{1}+(1+v_{s})v_{s}H_{2}}$$

where, the parameters *w* and *b* represent the dimensions of the width and the thickness of the micro-beam, respectively. And:

$$\begin{array}{c} H_{1}=\frac{2}{3}w^{3}-\frac{2}{3}{\left(b^{2}+w^{2}\right)}^{\overset{3}{\;}/\underset{2}{\;}}+\frac{2}{3}b^{3}+b^{2}w\ln(\frac{\sqrt{b^{2}+w^{2}}+w}{\sqrt{b^{2}+w^{2}}-w})+bw^{2}\ln(\frac{\sqrt{b^{2}+w^{2}}+b}{\sqrt{b^{2}+w^{2}}-b}),\\ H_{2}=\frac{4}{3}b^{3}-\frac{2}{3}w^{3}+2w^{2}\sqrt{\left(b^{2}+w^{2}\right)}-\frac{4}{3}{\left(b^{2}+w^{2}\right)}^{\overset{3}{\;}/\underset{2}{\;}}+b^{2}w\ln(\frac{\sqrt{b^{2}+w^{2}}+w}{\sqrt{b^{2}+w^{2}}-w}). \end{array}$$

### 2.2. The Rotational Equivalent Stiffness

**Figure 1.**
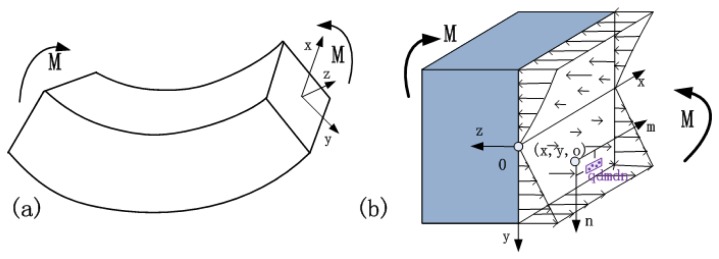
(**a**) The deformation of the rectangular cross-section beam in pure bending; (**b**) Non-uniform normal stress acting on the cross-section of the beam supports.

When the bending moment is acting on the beam, the non-uniform normal stress is correspondingly applied on the support, as shown in the rectangular region (*b* × *w*) of Figure 1b. In the presented model, the strain distribution based on small deflection theory was studied using the theories of beam bending. The curvature of the deflection curve is small under small deformation conditions. The slope equation and deflection equation are linear functions of the load. During the derivation of Euler's formula, the precise curvature $\frac{d^{2}y/dx^{2}}{{\left[1+{\left(d^{2}y/dx^{2}\right)}^{2}\right]}^{\overset{3}{\;}/\underset{2}{\;}}}$ is replaced by the approximation curvature $\frac{d^{2}y}{dx^{2}}$ due to the small deflection theory. Moreover, according to the elastic mechanics theory, the shear effect on the distribution and the maximum value of the normal stress is usually less than 5% as the ratio of beam span and thickness is greater than 5. Thus, we simply assumed that the linear stress distribution near the supports for pure bending was also able to meet the hypotheses and approximations related to the Boussinesq's and Cerruti's solutions.

The displacement of any point (*x*, *y*) in the normal direction due to the force of the differential unit *dp* = *σ_z_dmdn* acting on the surface can be obtained from the Boussinesq's displacement equations [47–49]:

(3) $$du(x,y)=\frac{1-v_{s}^{2}}{\pi E_{s}}\frac{dP}{\sqrt{x^{2}+y^{2}}}=\frac{1-v_{s}^{2}}{\pi E_{s}}\frac{\sigma_{z}(x,y)\text{dmdn}}{\sqrt{{\left(m-x\right)}^{2}+{\left(n-y\right)}^{2}}}=\frac{(1-v_{s}^{2})M_{x}}{\pi E_{s}I_{x}}\frac{\text{ndmdn}}{\sqrt{{\left(m-x\right)}^{2}+{\left(n-y\right)}^{2}}}$$

where *M_x_* is the bending moment applied to the supports, *I_x_* is the moment of inertia for the cross-section of the support. The normal stress *σ_z_* of the cross-section of the support is proportional to the bending moment. In the same way, utilizing the superposition principle and the energy equivalence principle, the equivalent bending stiffness of the flexible support had been derived as:

(4) $$K_{R}=\frac{M_{x}}{\bar{\theta }}=\frac{M_{x}^{2}}{U_{b}}=\frac{2\pi E_{s}I_{x}^{2}}{(1-v_{s}^{2})ℜ}$$

where:

$$\begin{array}{l} ℜ=\iint_{w\times b}\text{yHdxdy}=\int_{-w/2}^{w/2}\int_{0}^{b}\text{yHdxdy}=\frac{1}{2}{\text{w}}^{3}{\text{b}}^{2}\ln\left(\frac{\text{w}+\sqrt{{\text{b}}^{2}+{\text{w}}^{2}}}{b}\right)+\frac{1}{2}{\text{w}}^{4}\text{b}\ln\left(\frac{4{\text{w}}^{3}}{\sqrt{{\text{b}}^{2}+{\text{w}}^{2}}+\text{b}}\right)\\ \;-{\text{b}}^{2}{\text{w}}^{2}{\left({\text{b}}^{2}+{\text{w}}^{2}\right)}^{1/2}+\frac{1}{6}{\text{w}}^{2}{\left({\text{b}}^{2}+{\text{w}}^{2}\right)}^{3/2}-\frac{2}{15}{\left({\text{b}}^{2}+{\text{w}}^{2}\right)}^{5/2}+\frac{2}{15}{\text{b}}^{5}-\frac{1}{30}{\text{w}}^{5}-\frac{1}{2}{\text{bw}}^{4}+\frac{7}{6}{\text{b}}^{3}{\text{w}}^{2}. \end{array}$$

### 2.3. The Comparison and Validation

The effect of non-ideal boundary conditions on the dynamics of the arch was investigated by Alkharabsheh *et al.* [27]. To match the experimentally measured natural frequency, rotational and transverse springs have been added to each end of the arch model. The values of stiffness coefficients are tuned when solving the eigenvalue problem, until theoretical and experimental values of the natural frequencies are matched. Table 1 lists the comparison of equivalent stiffness coefficients between the proposed results and the experimental data from Alkharabsheh *et al.* [27]. It can be found that the proposed results close to the experimental data [27], which are calculated from Equation (10) and the testing data (*α_t_* and *α_R_*).

A similar experiment for the atomic force microscope (AFM) micro-cantilever probes was presented by Rinaldi *et al*. [41]. The work provided a testing method in which most of the influences were quantified or made variant while the supports condition being kept invariant. The testing data from [41] and the calculated results are listed in Table 2. It shows that the proposed results follow the evaluation approach presented by Rinaldi *et al.* [41]. As there is no translational motion at the support, the value of *K_t_* should be maintained at a high value. Therefore, the difference of the translation stiffness coefficients between the presented results and the experimental data [41] has not difficult to understand.

## 3. Dynamical Model and Analysis

**Figure 2.**
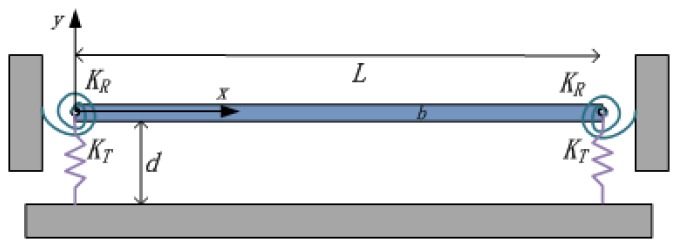
Schematic representation of an electrically actuated beam with compliant supports.

In this section, we formulate the problem for the forced vibration of a microbeam of nonideal supports. Rotational and transversal springs are added to the boundaries of the beam to model the compliant supports shown schematically in Figure 2. Assuming Euler-Bernoulli beam model with immovable end conditions causing nonlinear stretching effects, the nonlinear equation of motion governing the transverse deflection of the beam is expressed as:

(5) $$\begin{array}{c} E_{b}I\frac{\partial^{4}\hat{w}(\hat{x},\hat{t})}{\partial {\hat{x}}^{4}}+\rho_{b}A\frac{\partial^{2}\hat{w}(\hat{x},\hat{t})}{\partial {\hat{t}}^{2}}+\frac{E_{b}A}{2L}\frac{\partial^{2}\hat{w}(\hat{x},\hat{t})}{\partial {\hat{x}}^{2}}\int_{0}^{L}{\left(\frac{\partial \hat{w}(\hat{x},\hat{t})}{\partial \hat{x}}\right)}^{2}d\hat{x}\\ +\tilde{c}\frac{\partial \hat{w}(\hat{x},\hat{t})}{\partial \hat{t}}=\tilde{F}\cos(\tilde{\Omega }t) \end{array}$$

where *ŵ*(*x̂*,*t̂*) is the transverse deflection, *x̂* is the spatial coordinate, *t̂* is time, *A* is the cross section, *I* is the area moment of inertia. *E_b_*, *ρ_b_* and *ν_b_* are the Young's modulus, density and Poisson's ratio of the beam material. *c˜* is the damping coefficient. *F˜* and Ω are the magnitude and frequency external excitation respectively. In Equation (5), the boundary conditions of the beam are:

(6) $$\begin{array}{cc} E_{b}I\frac{\partial^{2}\hat{w}(0,\hat{t})}{\partial {\hat{x}}^{2}}-K_{R}\frac{\partial \hat{w}(0,\hat{t})}{\partial \hat{x}}=0, & -E_{b}I\frac{\partial }{\partial \hat{x}}(\frac{\partial^{2}\hat{w}(0,\hat{t})}{\partial {\hat{x}}^{2}})-K_{T}\hat{w}(0,\hat{t})=0\\ E_{b}I\frac{\partial^{2}\hat{w}(L,\hat{t})}{\partial {\hat{x}}^{2}}+K_{R}\frac{\partial \hat{w}(L,\hat{t})}{\partial \hat{x}}=0, & E_{b}I\frac{\partial }{\partial \hat{x}}(\frac{\partial^{2}\hat{w}(L,\hat{t})}{\partial {\hat{x}}^{2}})-K_{T}\hat{w}(L,\hat{t})=0 \end{array}$$

For convenience, the following nondimensional variables are introduced:

(7) $$\begin{array}{ccc} w=\hat{w}/d, & x=\hat{x}/L, & t=\hat{t}/T \end{array}$$

where *T* is a time constant defined by $T=\sqrt{\rho_{b}A/E_{b}IL^{4}}$. The natural frequency is defined as $\omega_{n}=\sqrt{EI/\rho A}/L^{2}$. Substituting the normalized variables of Equation (7) into Equations (5 and (6) yields the following non-dimensional equation of motion and boundary conditions:

(8) $$\frac{\partial^{4}w(x,t)}{\partial x^{4}}+\frac{\partial^{2}w(x,t)}{\partial t^{2}}+\alpha_{1}\frac{\partial^{2}w(x,t)}{\partial x^{2}}{\int_{0}^{1}\left(\frac{\partial w(x,t)}{\partial x}\right)}^{2}dx+c\frac{\partial w(x,t)}{\partial t}=F\cos(\Omega t)$$

(9) $$\begin{array}{cc} \frac{\partial^{2}w(0,t)}{\partial x^{2}}=\alpha_{R}\frac{\partial w(0,t)}{\partial x}, & \frac{\partial^{3}w(0,t)}{\partial x^{3}}=-\alpha_{T}w(0,t)\\ \frac{\partial^{2}w(1,t)}{\partial x^{2}}=-\alpha_{R}\frac{\partial w(1,t)}{\partial x}, & \frac{\partial^{3}w(1,t)}{\partial x^{3}}=\alpha_{T}w(1,t) \end{array}$$

The nondimensional parameters in Equations (8) and (9) are defined as:

(10) $$\begin{array}{llllll} \alpha_{1}=6{\left(\frac{d}{h}\right)}^{2}, & F=\frac{\tilde{F}L^{4}}{E_{b}Id}, & \Omega =\frac{\tilde{\Omega }}{\omega_{n}}, & c=\frac{\tilde{c}L^{4}}{E_{b}IT}, & \alpha_{T}=\frac{K_{T}L^{3}}{E_{b}I}, & \alpha_{R}=\frac{K_{R}L}{E_{b}I} \end{array}$$

### 3.1. The Resonance Frequency and Mode Shape Analysis

First, we study the effect of non-ideal boundary conditions on the resonance frequencies and mode shapes of the beam. These springs affect the stiffness of beam and, hence, its frequencies and mode shapes. The linearized undamped and unforced version of Equation (8) can be obtained by dropping the forcing and damping terms and considering only the linear terms in *w(x, t)*, yields:

(11) $$w^{\prime \prime \prime \prime }(x,t)+\ddot{w}(x,t)=0$$

where the superscript “prime” and “dot”, respectively, mean derivative with respect to *x* and *t*. We use separation of variables, and assume:

(12) $$w(x,t)=\phi (x)e^{i\omega t}$$

where *ϕ(x)* is the assumed mode shape and *ω* is the corresponding natural frequency. Substituting Equation (12) into Equations (11) and (9) yields:

(13) $$\phi^{\prime \prime \prime \prime }(x)-\omega^{2}\phi (x)=0$$

(14) $$\begin{array}{cc} \phi^{\prime \prime }(0)=\alpha_{R}\phi^{\prime }(0), & \phi^{\prime \prime \prime }(0)=\alpha_{T}\phi (0)\\ \phi^{\prime \prime }(1)=-\alpha_{R}\phi^{\prime }(1), & \phi^{\prime \prime \prime }(1)=\alpha_{T}\phi (1) \end{array}$$

The homogeneous solution of this fourth-order ordinary differential equation can be expressed as:

(15) $$\phi (x)=a_{1}\cos(\sqrt{\omega }x)+a_{2}\sin(\sqrt{\omega }x)+a_{3}\cosh(\sqrt{\omega }x)+a_{4}\sinh(\sqrt{\omega }x)$$

where *a_i_* (*i* = 1,2,3,4) are integration constants. The eigenvalue problem can be established by applying the boundary conditions of Equation (14) on Equation (15), which gives an algebraic system of equations to be solved for the natural frequencies:

(16) $$\left|\begin{array}{cccc} \sqrt{\omega } & \alpha_{R} & -\sqrt{\omega } & \alpha_{R}\\ \alpha_{T} & -\omega^{3/2} & \alpha_{T} & \omega^{3/2}\\ \Psi_{31} & \Psi_{32} & \Psi_{33} & \Psi_{34}\\ \Psi_{41} & \Psi_{42} & \Psi_{43} & \Psi_{44} \end{array}\right|=0$$

where:

$$\begin{array}{ll} \Psi_{31}=-\sqrt{\omega }\cos\sqrt{\omega }-\alpha_{R}\sin\sqrt{\omega }, & \Psi_{32}=-\sqrt{\omega }\sin\sqrt{\omega }+\alpha_{R}\cos\sqrt{\omega }\\ \Psi_{33}=\sqrt{\omega }\cosh\sqrt{\omega }+\alpha_{R}\sinh\sqrt{\omega }, & \Psi_{34}=\sqrt{\omega }\sinh\sqrt{\omega }+\alpha_{R}\cosh\sqrt{\omega }\\ \Psi_{41}=\omega^{3/2}\sin\sqrt{\omega }-\alpha_{T}\cos\sqrt{\omega }, & \Psi_{42}=-\omega^{3/2}\cos\sqrt{\omega }-\alpha_{T}\sin\sqrt{\omega }\\ \Psi_{43}=\omega^{3/2}\sinh\sqrt{\omega }-\alpha_{T}\cosh\sqrt{\omega }, & \Psi_{44}=\omega^{3/2}\cosh\sqrt{\omega }-\alpha_{T}\sinh\sqrt{\omega } \end{array}$$

*ω_i_* (*i* = 1,2,3,…) are obtained by numerical solution, and $\omega_{0i}=\omega_{i}\sqrt{EI/\rho A}/L^{2}(i=1,2,3,\ldots ).$ Meanwhile, the corresponding mode shapes are obtained as:

(17) $$\begin{array}{c} \phi_{i}(x)=\frac{\alpha_{T}\alpha_{R}-\omega_{i}^{2}}{2\alpha_{T}\sqrt{\omega_{i}}}[\chi_{i}\cosh(\sqrt{\omega_{i}}x)-\cos(\sqrt{\omega_{i}}x)]+\sin(\sqrt{\omega_{i}}x)\\ +\frac{\alpha_{T}\alpha_{R}+\omega_{i}^{2}}{2\alpha_{T}\sqrt{\omega_{i}}}[\cosh(\sqrt{\omega_{i}}x)-\chi_{i}\cos(\sqrt{\omega_{i}}x)]+\chi_{i}\sinh(\sqrt{\omega_{i}}x) \end{array}$$

where:

$$\chi_{i}=\frac{\begin{array}{l} (3\omega_{i}^{2}-\alpha_{T}\alpha_{R})\alpha_{T}\cos\sqrt{\omega_{i}}+\left(\alpha_{T}\alpha_{R}\omega_{i}^{1.5}-\omega_{i}^{3.5}+2\alpha_{T}^{2}\sqrt{\omega_{i}}\right)\sin\sqrt{\omega_{i}}\\ \;+(\omega_{i}^{2}+\alpha_{T}\alpha_{R})\alpha_{T}\cosh\sqrt{\omega_{i}}-\left(\alpha_{T}\alpha_{R}\omega_{i}^{1.5}+\omega_{i}^{3.5}\right)\sinh\sqrt{\omega_{i}} \end{array}}{\begin{array}{l} (\omega_{i}^{2}+\alpha_{T}\alpha_{R})\alpha_{T}\cos\sqrt{\omega_{i}}-\left(\alpha_{T}\alpha_{R}\omega_{i}^{1.5}+\omega_{i}^{3.5}\right)\sin\sqrt{\omega_{i}}\\ \;+(3\omega_{i}^{2}-\alpha_{T}\alpha_{R})\alpha_{T}\cosh\sqrt{\omega_{i}}+\left(\alpha_{T}\alpha_{R}\omega_{i}^{1.5}-\omega_{i}^{3.5}-2\alpha_{T}^{2}\sqrt{\omega_{i}}\right)\sinh\sqrt{\omega_{i}}. \end{array}}$$

The equivalent tangential stiffness and equivalent rotational stiffness of the flexible supports are listed in the second and third columns of Table 3. It is interestingly found that there are large differences of the equivalent stiffness between the two different types of supports material (silicon carbide and polysilicon). If the ratio between the Young's modulus and density of the material is large, we define the material as a “hard material”. On the contrary, a “soft material” is defined when the ratio is small [50]. From Table 3, it can be found that when a “hard material”, e.g., silicon carbide, is used as the support material, the equivalent stiffness is large, while the equivalent stiffness is small for a “soft material”, e.g., polysilicon.

**Figure 3.**
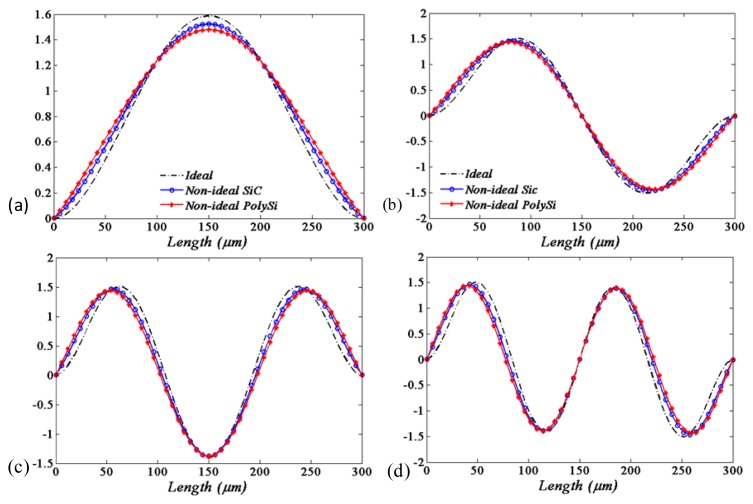
First four mode shapes corresponding to the natural frequencies of Table 3. ((Dashed) Ideal boundary conditions, (blue circle), (red star) non-ideal boundary conditions with the supports material of silicon carbide and polysilicon, respectively). (**a**) First; (**b**) Second; (**c**) Third; (**d**) Fourth.

By solving the eigenvalue problem of Equation (16), the first four values of new natural frequencies of the beam in the presence of the springs are obtained, as shown in the fourth to seventh columns of Table 3. It demonstrates that the natural frequencies of the beam will be smaller when considering the flexible boundary conditions. It is also shown that the natural frequencies with the “soft material” (polysilicon) flexible supports conditions will be smaller compared with those under the “hard material” (silicon carbide) supports conditions. The corresponding mode shapes compared to those of ideal boundary conditions are shown in Figure 3.

It is obvious that the qualitative and quantitative behaviors of the mode shape are different for different boundary conditions. The amplitude of the ideal supports is less than the flexible one as the position close the substrate. However, the situation is almost reversed as the position away from the substrate. Moreover, this trend will be more strengthen as the softer material (polysilicon) is used.

It is also easily observed that the actual modal is more close to the ideal modal when the supports' material performance approximates to the rigid body. Moreover, the softer the supports' material is, the greater the difference between the actual modals and ideal modals will be.

### 3.2. Frequency-Response Analysis

Next, we study the effect of the nonideal boundary conditions on the dynamics response of the beam. To solve the Equation (8), two time scales *T_0_* = *t*, *T_1_* = *εt* are introduced [52], and a first-order uniform approximate solution is given in the form:

(18) $$w\left(x,\tau ;ɛ\right)=w_{0}\left(x,T_{0},T_{1}\right)+ɛw_{1}\left(x,T_{0},T_{1}\right)+\cdots$$

where *T_0_* is the usual fast time scale and *T_1_* = *εt* is the slow time scale in the method of multiple scales. The time derivatives are defined as:

(19) $$\begin{array}{cc} \text{d}/\text{d}\tau =D_{0}+ɛD_{1}+\cdots , & {\text{d}}^{2}/\text{d}\tau^{2}=D_{0}^{2}+2ɛD_{0}D_{1}+\cdots \end{array}$$

where D_0_=∂/∂*T*_0_, D_1_=∂/∂*T*_1_. By introducing the following variables *α*_1_=*εα̂*_1_, *c*=2*εμ*, *F*=*εF̂*, *α_T_*=*εα̂_T_*, *α_R_*=*εα̂_R_*, where *ε* is a small perturbation parameter denoting that the variations are small, and substituting Equations (18) and (19) into Equations (8) and (9), equating coefficients of like powers of *ε* yields for order *ε^0^* and order *ε^1^*, we get a set of linear partial differential equations:

(20) $$\begin{array}{lll} {ɛ}^{0} & {\text{D}}_{0}^{2}w_{0}+w_{0}^{iv}=0, & \\ & w_{0}^{\prime \prime }(0,T_{0},T_{1})=0, & w_{0}^{\prime \prime \prime }(0,T_{0},T_{1})=0,\\ & w_{0}^{\prime \prime }(1,T_{0},T)=0, & w_{0}^{\prime \prime \prime }(1,T_{0},T_{1})=0. \end{array}$$

(21) $$\begin{array}{ll} {ɛ}^{1} & {\text{D}}_{0}^{2}w_{1}+w_{1}^{iv}=-2\mu {\text{D}}_{0}w_{0}-2{\text{D}}_{0}{\text{D}}_{1}w_{0}-{\hat{\alpha }}_{1}w_{0}^{\prime \prime }\int_{0}^{1}{\left(w_{0}^{\prime }\right)}^{2}dx+\hat{F}\cos(\Omega T_{0})\\ & \begin{array}{cc} w_{1}^{\prime \prime }(0,T_{0},T_{1})={\hat{\alpha }}_{R}w_{0}^{\prime }(0,T_{0},T_{1}), & w_{1}^{\prime \prime \prime }(0,T_{0},T_{1})=-{\hat{\alpha }}_{T}w_{0}(0,T_{0},T_{1}) \end{array}\\ & \begin{array}{cc} w_{1}^{\prime \prime }(1,T_{0},T)=-{\hat{\alpha }}_{R}w_{0}^{\prime }(1,T_{0},T), & w_{1}^{\prime \prime \prime }(1,T_{0},T_{1})={\hat{\alpha }}_{T}w_{0}(1,T_{0},T_{1}) \end{array} \end{array}$$

The general solution of first equation of Equation (20) can be written as:

(22) $$w_{0}\left(T_{0},T_{1}\right)=\text{a}\left(T_{1}\right)\cos\left[\omega T_{0}+\delta (T_{1})\right]\text{Y}(\text{x})=(A(T_{1})\exp^{j\omega T_{0}}+cc\text{Y}(\text{x})$$

where *A*(*T*_1_)=a(*T*_1_)/2*e^jβ^*^(^*^T^*^1)^, and cc denotes complex conjugate. Substituting (22) into (20) yields the boundary value problem:

(23) $${\text{Y}}^{iv}-\omega^{2}\text{Y}=0,$$

(24) $$Y^{\prime \prime }(0)=Y^{\prime \prime \prime }(0)=Y^{\prime \prime }(1)=Y^{\prime \prime \prime }(1)=0$$

The solution is:

(25) $$\begin{array}{cc} \cos\sqrt{\omega_{i}}.\cosh\sqrt{\omega_{i}}=1, & \left(i=1,2,3,\ldots \right) \end{array}$$

(26) $$Y_{i}(x)=\chi_{i}[\cos(\sqrt{\omega_{i}}x)+\cosh(\sqrt{\omega_{i}}x)]+\sin(\sqrt{\omega_{i}}x)+\sinh(\sqrt{\omega_{i}}x)$$

where $\chi_{i}=-\left(\sin(\sqrt{\omega_{i}})-\sinh(\sqrt{\omega_{i}})\right)/\left(\cos(\sqrt{\omega_{i}})-\cosh(\sqrt{\omega_{i}})\right)=\left(\cos(\sqrt{\omega_{i}})-\cosh(\sqrt{\omega_{i}})\right)/\left(\sin(\sqrt{\omega_{i}})+\sinh(\sqrt{\omega_{i}})\right)$, and *Y_i_(x)* is normalized such that $\int_{0}^{1}Y_{\text{i}}Y_{\text{j}}dx=\delta_{ij}$.

At order *ε*, one substitutes (22) into the right hand side of (21). The result is:

(27) $${\text{D}}_{0}^{2}w_{1}+w_{1}^{iv}=[-2i\omega {\text{D}}_{1}AY-2i\mu \omega AY-3{\hat{\alpha }}_{1}A^{2}\bar{A}Y^{\prime \prime }\int_{0}^{1}{\left(Y^{\prime }\right)}^{2}dx+\hat{F}/2\exp^{i\sigma T_{1}})]\times \exp^{i\omega T_{0}}+\text{NSY}+cc$$

where NSF stands for non-secular terms. It is assumed that the external excitation frequency is close to one of the natural frequencies of the system:

(28) Ω=ω+ɛσ

Where σ is a detuning parameter of order 1. A solution of the form is assumed as:

(29) $$w_{1}=\varphi (x,T_{1})\exp^{i\omega T_{0}}+W_{1}(x,T_{0},T_{1})+cc$$

The first part of the solution is the one corresponding to secular terms and the second is the one corresponding to non-secular terms. Substituting Equation (29) into Equation (27) with boundary conditions yields:

(30) $$\varphi^{iv}-\omega^{2}\varphi =-2i\omega {\text{D}}_{1}AY-2i\mu \omega AY-3{\hat{\alpha }}_{1}A^{2}\bar{A}Y^{\prime \prime }\int_{0}^{1}{\left(Y^{\prime }\right)}^{2}dx+\hat{F}/2\exp^{i\sigma T_{1}}$$

(31) $$\begin{array}{cc} \varphi^{\prime \prime }(0,T_{1})={\hat{\alpha }}_{R}A(T_{1}){Y^{\prime }}_{0}(0), & \varphi^{\prime \prime \prime }(0,T_{1})=-{\hat{\alpha }}_{T}A(T_{1})Y_{0}(0)\\ \varphi^{\prime \prime }(1,T_{1})=-{\hat{\alpha }}_{R}A(T_{1}){Y^{\prime }}_{0}(1), & \varphi^{\prime \prime \prime }(1,T_{1})={\hat{\alpha }}_{T}A(T_{1})Y_{0}(1) \end{array}$$

Since the homogeneous problem has a non-trivial solution, the non-homogeneous problem (30) and (31) have a solution only if a solvability condition is satisfied. Therefore, by multiplying *Y(x)* on both sides of Equation (30), and then integrating them from 0 to 1, the non-trivial solution can be obtained. Through combining the boundary conditions of Equations (24) and (31), it is found that:

(32) $$\begin{array}{l} \int_{0}^{1}\varphi^{iv}\text{Ydx}={\varphi^{\prime \prime \prime }Y|}_{0}^{1}-{\varphi^{\prime \prime }Y^{\prime }|}_{0}^{1}+{\varphi^{\prime }Y^{\prime \prime }|}_{0}^{1}-{\varphi Y^{\prime \prime \prime }|}_{0}^{1}+\int_{0}^{1}\varphi Y^{iv}dx\\ =\varphi^{\prime \prime \prime }(1)Y(1)-\varphi^{\prime \prime \prime }(0)Y(0)-\varphi^{\prime \prime }(1)Y^{\prime }(1)+\varphi^{\prime \prime }(0)Y^{\prime }(0)+\varphi^{\prime }(1)Y^{\prime \prime }(1)-\varphi^{\prime }(0)Y^{\prime \prime }(0)\\ \;-\varphi (1)Y^{\prime \prime \prime }(1)+\varphi (0)Y^{\prime \prime \prime }(0)+\omega^{2}\int_{0}^{1}\varphi \text{Ydx}\\ ={\hat{\alpha }}_{T}A[Y^{2}(1)-4\chi^{2}]-{\hat{\alpha }}_{R}A[{Y^{\prime }}^{2}(1)+4\omega ]+\omega^{2}\int_{0}^{1}\varphi \text{Ydx} \end{array}$$

So the solvability condition of Equations (30) and (31) requires:

(33) $$-\text{KKA}+2i\omega ({\text{D}}_{1}A+\mu A)+3{\hat{\alpha }}_{1}A^{2}\bar{A}ℜℜ-\hat{F}/2\exp^{i\sigma T_{1}}=0$$

where:

$$\begin{array}{l} KK:={\tilde{\alpha }}_{T}[4\chi^{2}-{\left[\chi (\cos\sqrt{\omega }+\cosh\sqrt{\omega })+\sin\sqrt{\omega }x+\sinh\sqrt{\omega }\right]}^{2}]\\ \;+\omega {\tilde{\alpha }}_{R}[4+{\left[\chi (-\sin\sqrt{\omega }x+\sinh\sqrt{\omega })+\cos\sqrt{\omega }+\cosh\sqrt{\omega }\right]}^{2}] \end{array}$$

$$\begin{array}{l} ℜℜ:=\int_{0}^{1}YY^{\prime \prime }\int_{0}^{1}{\left(Y^{\prime }\right)}^{2}dxdx\\ =\frac{\sqrt{\omega }}{4}\exp^{-4\sqrt{\omega }}\times \{[2\chi +2\sqrt{\omega }-\chi \cos2\sqrt{\omega }+1/2(\chi^{2}-1)\sin2\sqrt{\omega }{]\text{e}}^{2\sqrt{\omega }}-1/4{\left(\chi +1\right)}^{2}{\text{e}}^{4\sqrt{\omega }}+1/4{\left(\chi -1\right)}^{2}\}\\ \;\times \{[7\chi -2\sqrt{\omega }+(\cos\sqrt{\omega }+2\cosh\sqrt{\omega })((\chi^{2}-1)\sin\sqrt{\omega }-2\chi \cos\sqrt{\omega })-2(\chi^{2}+1)\cos\sqrt{\omega }.\sinh\sqrt{\omega }]\exp^{2\sqrt{\omega }}\\ \;-1/4{\left(\chi +1\right)}^{2}{\text{e}}^{4\sqrt{\omega }}+1/4{\left(\chi -1\right)}^{2}\} \end{array}$$

Then by expanding the trigonometric functions, and separating real and imaginary parts, the secular terms yields two first order nonlinear ordinary-differential equations that describe the amplitude *α* and phase *β* modulation of the response:

(34) $$\begin{array}{l} \omega {\text{D}}_{1}a=-\omega \mu a+\frac{1}{2}\hat{F}\sin\gamma \\ \omega a{\text{D}}_{1}\gamma =\sigma \omega a+\frac{1}{2}\text{KKa}-\frac{3}{8}{\hat{\alpha }}_{1}a^{3}ℜℜ+\frac{1}{2}\hat{F}\cos\gamma \end{array}$$

where *γ*=*σT*_1_−*β*. The steady-state motions occur when D_1_a=D_1_*γ*=0, which corresponds to singular points of Equation (34):

(35) $$\begin{array}{l} \mu \bar{a}=\frac{\hat{F}}{2\omega }\sin\bar{\gamma }\\ \sigma \bar{a}+\frac{KK}{2\omega }\bar{a}-\frac{3{\hat{\alpha }}_{1}ℜℜ}{8\omega }{\bar{a}}^{3}=-\frac{\hat{F}}{2\omega }\cos\bar{\gamma } \end{array}$$

Therefore, a function of the independent variables can be given by:

(36) $$\Omega =\omega -\frac{ɛKK}{2\omega }+3\frac{\alpha_{1}ℜℜ}{8\omega }{\bar{a}}^{2}\pm \frac{1}{2}\sqrt{\frac{F^{2}}{4\omega^{2}a^{2}}-c^{2}}$$

**Figure 4.**
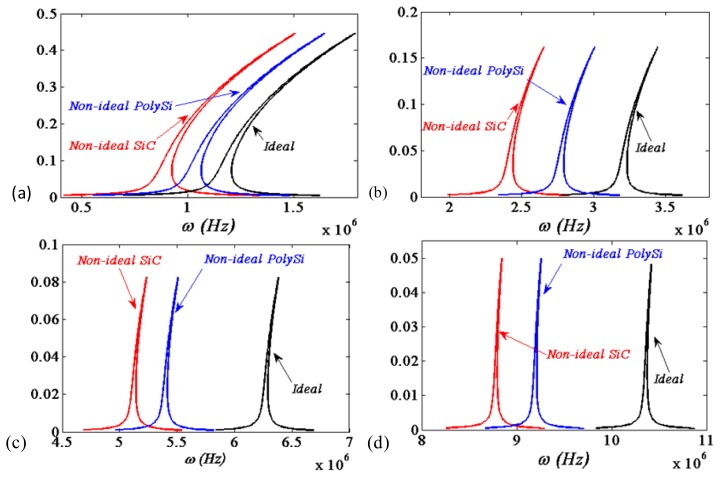
First four resonance response curves. (Black) Ideal boundary conditions. (blue), (red) non-ideal boundary conditions with the supports material of silicon carbide and polysilicon, respectively. (**a**) First; (**b**) Second; (**c**) Third; (**d**) Fourth.

In Figure 4, the first four frequency response curves are compared between the ideal and non-ideal boundary condition cases as *c* = *0.6*, *f* = *4*, respectively. The polysilicon microbeam has the material properties: Young's modulus *E* = 150 G*Pa*, density *ρ* = 2.3 × 10^3^*kg/m^3^* and Poisson's ratio *ν_s_* = 0.226. It is shown that small variations of deflections and moments at the ends would affect the frequencies of the response, deviations from the ideal conditions lead to a drift in the frequency-response curves.

From Equations (2) and (4), it is known that the softer the support material is, the smaller the equivalent stiffness will be. Furthermore, it is easily observed from Figure 4 that the smaller the equivalent stiffness is, the smaller the resonance frequencies of the MEMS beam resonators will be.

In the published literatures [19–21,26,30], Boyaci *et al.* studied the ideal and non-ideal as well as frequency response curves using the method of multiple scales. Their results shown that small variations of deflections and moments at the support ends affected the response frequencies. The values of the dimensionless spring stiffness of *K_T_* and *K_R_* in their article are set to represent different cases. The frequencies may increase or decrease depending on the mode numbers and amplitudes of variations. Deviations from the ideal conditions lead to a drift in frequency response curves which may be positive, negative or zero depending on the mode number and amplitudes of variations.

However, with the equivalent stiffness we have derived, it is found from Figure 4 that the drift in frequency response curves all are negative no matter what the mode number and amplitudes of vibrations are. This is a new finding different from the existing conclusions of Boyaci *et al.* [19–21,26,30].

## 4. Results and Discussion

The first four natural frequencies and mode shapes of the beam are obtained using the finite element software under rigid and flexible boundary conditions, respectively.

**Figure 5.**
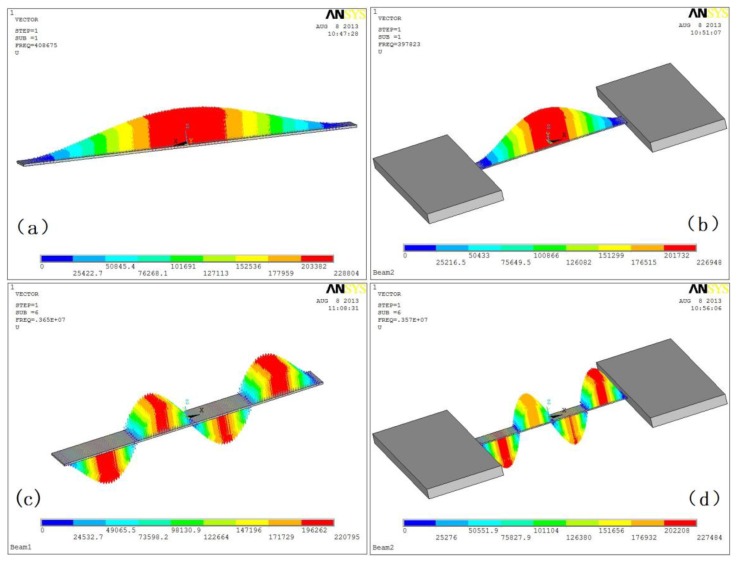
Modal vectors of the first and fourth mode shapes solved by FEM. (**a**) and (**b**): First modal under rigid and flexible boundary, respectively; (**c**) and (**d**): Fourth modal under rigid and flexible boundary, respectively.

The results were list in Table 4 and contrasted with the proposed results which list in Table 3. It is clear that the proposed analytical solutions are in good agreement with the finite element results. The natural frequencies of the beam under the flexible boundary conditions are smaller than the ones under rigid boundary conditions. Moreover, the corresponding modal vectors of the first and fourth mode shapes are shown in Figure 5.

It can be easily found from Figure 5b,d that there are small displacements on the support boundary, in addition, the displacements in the fourth modal are larger than the ones in the first modal. But there isn't any displacement when the rigid supporting conditions are applied on the beam, which is shown in Figure 5a,c. Moreover, the peak value of the mode shape under rigid supports is larger than the ones under flexible supports.

**Figure 6.**
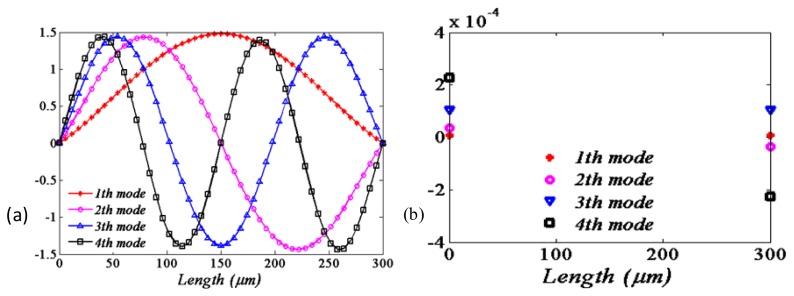
(**a**) First four mode shapes corresponding to the natural frequencies of Table 3; (**b**) The boundary displacement of each modal.

Figure 6a illustrates the first four mode shapes corresponding to the natural frequencies as listed in Table 5, where polysilicon is used as the support material. The partial enlarged figure of each mode is intercepted as shown in Figure 6b, where both ends displacements of the flexible supports boundary are displayed. The numerical results are also displayed in the Table 5. It is apparent that the small tangential displacements of both ends are symmetry with the odd mode number, while asymmetry with the even mode numbers. The tangential displacement becomes larger with the higher order modal.

The maximum amplitude of oscillation is reached when the magnitude under the square root in Equation (36) is zero. Hence, ā_max_=*F*/(2*ω*c). So the relation for the resonance frequency shift with respect to the maximum amplitude of oscillation can be derived as:

(37) $$\Omega =\omega -\frac{ɛKK}{2\omega }+\frac{3\alpha_{1}ℜℜ}{8\omega }{\bar{a}}_{\max}^{2}$$

**Figure 7.**
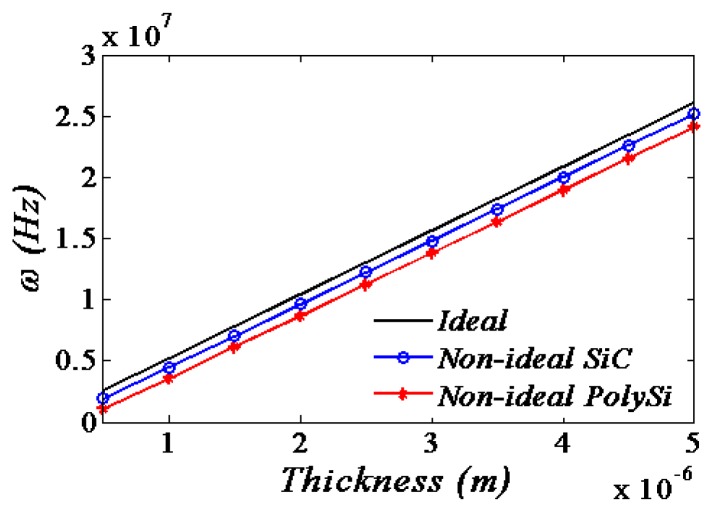
The resonant frequency shift to the beam thickness with respect to the different flexible supports materials.

In the above relation, the second term is due to the non-ideal boundary conditions and the third term is due to the nonlinearity mechanical spring. It can be obviously seen that the flexible supports conditions would decrease the resonance frequency while the nonlinearity mechanical spring increase the frequencies. However, it is also apparent of *KK* that rotational springs become dominant compared to transversal springs as the mode number increases. So it may increase or decrease the frequencies depending on the mode number *i* and the amplitudes of the supports variations [20]. Therefore, it is easy to infer the effects of the material performances and the geometric sizes of the supports conditions on the resonant frequency from Equation (37). The resonant frequency shift to the beam thickness with respect to the different flexible supports material is shown in Figure 7.

The resonance frequencies increase linearly with the beam thickness. Moreover, the resonance frequencies of the flexible resonators are smaller than the ones of the ideal support resonators no matter what value the beam thickness is.

In fact, it is revealed that the material performances and the geometric sizes of the supports conditions not only influence the system stiffness and the resonant frequency shift, but also affect the system vibration amplitude. That was presented in the paper [51] by considering the clamping loss due to the phonon tunneling.

## 5. Conclusions

In this paper, we have quantitatively studied the effect of the flexible supports boundary conditions on the dynamic characteristics of MEMS beams. Utilizing the tangential and rotational equivalent stiffness formulations derived by employing Cerruti's and Boussinesq's displacement equations and the principle of energy equivalence, rigorous theoretical dynamic analytical models are presented.

It is of great significance to investigate the rigorous variation of the resonant frequency and dynamic response due to the equivalent stiffness of the flexible supports, where the nonlinearity mechanical spring increases the frequencies while the flexible supports conditions decrease the resonance frequency. It is also demonstrated that the support material property has an important influence on the equivalent stiffness, dynamic response and the resonant frequency shift. The advantage of the proposed solution is that no approximated displacement and force fields are introduced during the derivation of the equivalent stiffness. Moreover, the proposed analytical solutions are in good agreement with the results obtained from finite element analyses. Based on the proposed solutions, it is convenient to quantitatively and accurately analyze the dynamics problem of the MEMS beams with the flexible support boundary conditions.

## Figures and Tables

**Table 1.** The comparison of the equivalent stiffness between the proposed results and the reported experimental data [27].

**The Comparison**	*K_R_* (10^−8^*N.m*/*rad*)
Experiment data 1 of Alkharabsheh *et al.* [27]	9.0396
Proposed results	7.4270
Experiment data 2 of Alkharabsheh *et al.* [27]	10
Proposed results	8.3001

**Table 2.** The comparison of the equivalent stiffness between the proposed results and the reported experimental data [41].

**The Comparison**	*K_t_* (*N/m*)	*K_R_* (10^−8^*N m/rad*)
Experiment data 1 of Rinaldi *et al.* [41]	9.8018 × 10^7^	13.284
Proposed results	1.9743 × 10^7^	16.567
Experiment data 2 of Rinaldi *et al.* [41]	1.6620 × 10^8^	13.521
Proposed results	1.9801 × 10^7^	17.120

**Table 3.** The equivalent tangential stiffness and equivalent rotational stiffness of the flexible supports and the first four natural frequencies of the beam. The second and third rows relate to the supports material [51] of silicon carbide (E = 415 GPa, density = 3,200 Kg/m^3^, v = 0.192) and polysilicon (E = 150 GPa, density = 2,300 Kg/m^3^, v = 0.226), respectively.

	***K****_T_***(10^6^N/m)**	***K****_R_***(10^−8^N.m/rad)**	***ω*_1_(10^6^Hz)**	***ω*_2_(10^6^Hz)**	***ω*_3_(10^6^Hz)**	***ω*_4_(10^6^Hz)**
Ideal boundary conditions	-	-	1.1591	3.1950	6.2635	10.354
Non-Ideal boundary conditions (SiC)	5.4996	17.895	0.95669	2.7104	5.4261	9.1206
Non-Ideal boundary conditions (PolySi)	1.9929	6.5648	0.80512	2.4305	5.0381	8.6479

**Table 4.** The first four natural frequencies of the beam solved by FEM.

**Boundary Conditions**	***ω*_1_(10^6^Hz)**	***ω*_2_(10^6^Hz)**	***ω*_3_(10^6^Hz)**	***ω*_4_(10^6^Hz)**
Rigid	1.0885	3.0365	6.0639	10.277
Flexible	0.9461	2.8851	5.8395	9.9402

**Table 5.** The boundary displacements of first four modal vectors.

**Displacement Positions**	**First Modal (10^−6^)**	**Second Modal (10^−6^)**	**Third Modal (10^−6^)**	**Fourth Modal (10^−6^)**
x = 0	6.9028	35.689	103.53	227.74
x = L	6.9028	−35.689	103.53	−227.74
